# The Role of the Disrupted Podosome Adaptor Protein (SH3PXD2B) in Frank–Ter Haar Syndrome

**DOI:** 10.3390/genes13020236

**Published:** 2022-01-27

**Authors:** Salam Massadeh, Fahad Alhabshan, Hadeel N. AlSudairi, Sarah Alkwai, Moneera Alsuwailm, Mohamed S. Kabbani, Farah Chaikhouni, Manal Alaamery

**Affiliations:** 1 Developmental Medicine Department, King Abdullah International Medical Research Center, King Saud Bin Abdulaziz University for Health Sciences, King Abdulaziz Medical City, Ministry of National Guard-Health Affairs (MNG-HA), Riyadh 11481, Saudi Arabia; massadehsa@ngha.med.sa (S.M.); hadeelnawaf98@gmail.com (H.N.A.); sarahalkwai@gmail.com (S.A.); moalsuwailm@gmail.com (M.A.); 2 KACST-BWH Centre of Excellence for Biomedicine, Joint Centers of Excellence Program, King Abdulaziz City for Science and Technology (KACST), Riyadh 11442, Saudi Arabia; 3 Saudi Human Genome Satellite Laboratory at King Abdulaziz Medical City, King Abdulaziz City for Science and Technology (KACST), Ministry of National Guard Health Affairs (MNGHA), Riyadh 11481, Saudi Arabia; 4 Department of Cardiac Sciences, King Abdullah International Medical Research Center, King Saud bin Abdulaziz University for Health Sciences, Ministry of the National Guard-Health Affairs, Riyadh 14611, Saudi Arabia; habshanf@ngha.med.sa (F.A.); kabbanim@ngha.med.sa (M.S.K.); chaikhounifa@NGHA.MED.SA (F.C.)

**Keywords:** *SH3PXD2B*, Frank–Ter Haar syndrome, missense point mutation, whole-exome sequencing

## Abstract

Frank–Ter Haar syndrome (FTHS), sometimes referred to as Ter Haar syndrome, is a rare hereditary disorder that manifests in skeletal, cardiac, and ocular anomalies, including hypertelorism, glaucoma, prominent eyes, and facial abnormalities. In this study, we performed whole-exome sequencing (WES) to identify the genetic component responsible for the phenotype of the index patient, a male infant born to a consanguineous family from Saudi Arabia. The analysis revealed a homozygous missense variant, c.280C>G, in the SH3PXD2B gene, which cosegregates with the familial phenotype with a plausible autosomal-recessive mode of inheritance, indicating a potential disease-causing association. The SH3PXD2B gene encodes a TKS4 podosome adaptor protein that regulates the epidermal growth factor signaling pathway. This study validates the critical function of the TKS4 podosome protein by suggesting a common mechanism underlying the pathogenesis of FTHS.

## 1. Introduction

Frank–Ter Haar syndrome (FTHS) is a rare hereditary condition associated with skeletal, cardiovascular, ocular, and craniofacial abnormalities [1,2]. In 1973, Frank et al. (1973) initially described an 18-month-old girl born to consanguineous parents who presented with skeletal dysplasia, ocular defect, dysmorphic facial features, as well as a global developmental delay (GDD). A decade later, Ter Haar reported a Dutch family consisting of three siblings with skeletal and craniofacial anomalies, in addition to severe cardiovascular complications [3]. The siblings’ presentation resembled an autosomal recessive form of the X-linked congenital disorder Melnick–Needles Syndrome [4]. Years later, because of the similarity between the Ter Haar and Frank phenotypes and their autosomal recessive inheritance patterns, the syndrome was defined as a separate entity and renamed Frank–Ter Haar syndrome [5].

A homozygosity mapping study that sought to localize the genetic basis of FTHS in 12 families with known or suspected consanguinity found that the most plausible location for the FTHS mutation is on chromosome 5q35.1 within the SH3PXD2B gene [6]. The authors’ analysis of FTHS patients from the 12 families identified five homozygous mutations in SH3PXD2B (one complete deletion of SH3PXD2B and four different intragenic mutations) in seven families, with no SH3PXD2B mutation detected in the remaining families [6]. The study findings also indicated that the mice lacked Tks4, a protein encoded by the SH3PXD2B gene, thus exhibiting similar craniofacial, skeletal, ocular, and cardiovascular defects to those observed in FTHS. This, in turn, demonstrates an association between the features of the syndrome and the gene itself [6]. To date, 40 patients have been reported to have FTHS [5], 20 of whom were found to carry mutations in SH3PXD2B [1].

This paper presents a case study of a baby with the FTHS phenotype who was homozygous for a variant of the SH3PXD2B gene, reported only once, thus making this the second report for this novel variant. We found that both the parents and a sibling were carriers for this variant. The index patient exhibited several classical features associated with FTHS.

## 2. Patients and Methods

### 2.1. Patient Description and Ethical Considerations

**Figure 1:**
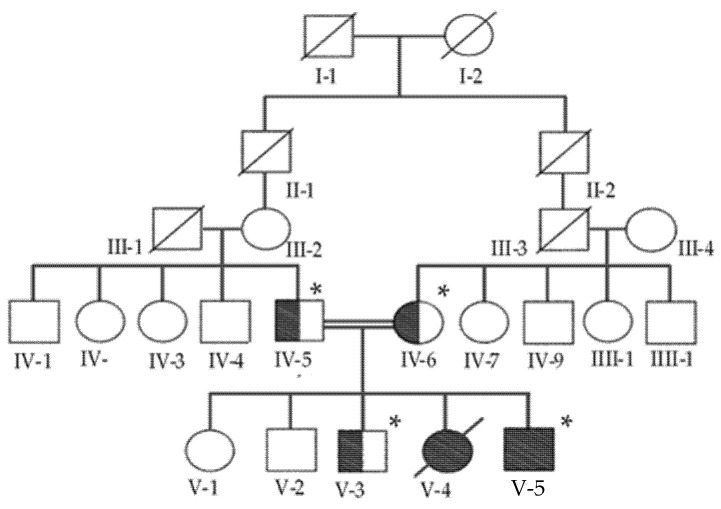
Family pedigree showcasing the degree of consanguinity, carrier, and affected individuals. The pedigree depicts an autosomal recessive mode of inheritance for this variant mutation. Double lines are indicative of a consanguineous union. The female and male individuals are represented with circles and square symbols, respectively. Filled symbols signify homozygous for the variant, while the half-filled symbols are heterozygous carriers. The individual numbers labeled with (asterisks) indicate the samples that were available for the study.

**Figure 2:**
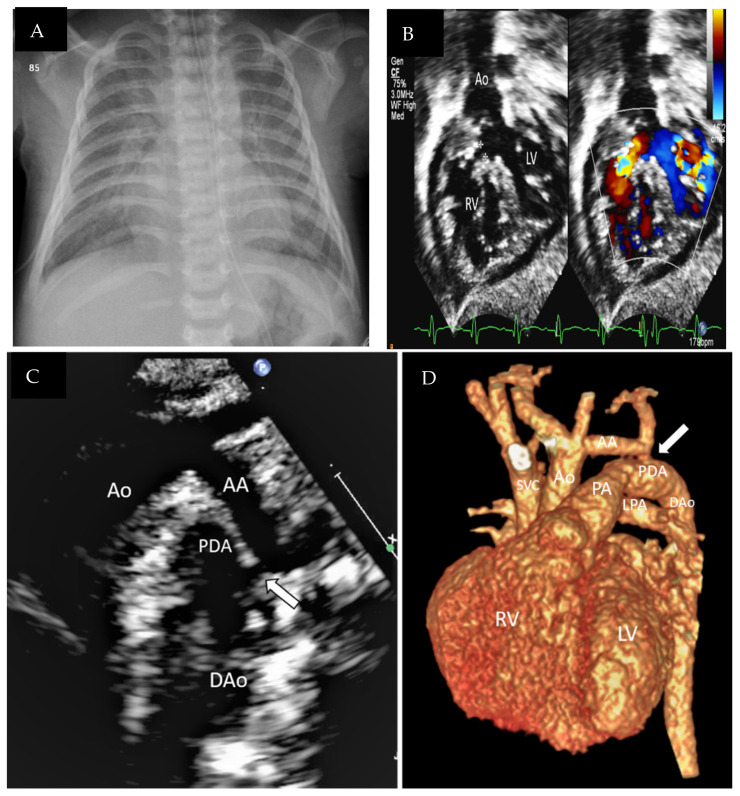
(**A**) Chest X-ray showing cardiomegaly and congested lungs. (**B**): Subcostal 2D and color compare echocardiographic view showing the subaortic VSD (* border of VSD). Ao, aorta; LV, left ventricle; RV, right ventricle. (**C**) Two-dimensional sagittal echocardiographic view of the aortic arch, and 3D reconstructed CT angiographic image (**D**) showing hypoplasia of the aortic arch with coarctation of the aorta (arrow) and a large PDA supplying the descending aorta. AA, aortic arch; Ao, aorta; Dao, descending aorta; LPA, left pulmonary artery; PA, pulmonary artery; PDA, patent ductus arteriosus; LV, left ventricle; RV, right ventricle; SVC, superior vena cava.

The index patient in this study was a <1-year-old boy who belonged to a consanguineous Saudi family (V-5, Figure 1). The family consisted of two healthy parents and five children (one affected male, two healthy males, one healthy female, and one deceased female). The index patient was diagnosed with severe coarctation (CoA) versus interrupted aortic arch (IAA), patent ductus arteriosus (PDA), ventricular septal defect (VSD), gastroesophageal reflux (GERD), and glaucoma. He was treated at the Department of Pediatric Cardiology, King Abdulaziz Medical City, Ministry of National Guard Health Affairs, Riyadh, Saudi Arabia. The deceased sister (V-4) reportedly had similar features to the index patient. One of the male siblings (V-3) and the parents (IV-5, IV-6) were carriers of the same variant mutation, whereas the remaining children (V-1, V-2) had no genetic abnormalities. Table 1 summarizes the index patient’s clinical features and medical history, and Figure 2 shows the chest X-ray and echocardiograms of the index patient. This study was approved by the Institutional Review Board Committee at King Abdullah International Medical Research Centre, Riyadh, and the patient’s parents provided written consent.

### 2.2. Genomic DNA Extraction

To extract DNA from blood samples, we used a QIAamp DNA Micro kit (Hilden, Germany). Then, a NanoDropTM spectrophotometer was used for DNA quantification and quality assessment.

### 2.3. Whole-Exome Sequencing (WES)

WES analysis of the proband (V-5), the healthy sibling (V-3), and both parents (IV-5, IV-6) was conducted by WES Centogene (Centogene GmbH, Rostock, Germany) using an Illumina platform (Illumina, Inc., San Diego, CA, USA). DNA fragmentation was performed with SureSelect Human All Exon V6 (Agilent Technologies, Santa Clara, CA, USA). The constructed library was sequenced to yield an average depth of coverage of 100*, and approximately 97% of the targeted bases were covered more than ten times.

### 2.4. Variant Annotation and Filtering

Sequence analysis was conducted via a sophisticated bioinformatics pipeline that encompasses applications such as base-calling and alignment of the reads to the GRCh37/hg19 genome assembly (GRCh37; http://genome.ucsc.edu/ (accessed on 29 October 2020)). A filtering process was performed to eliminate low-quality reads and potential artifacts, followed by the annotation of variants. Detected variants were screened against various databases, including the Human Mutations Database (www.hgmd.cf.ac.uk/ac/index.php, the Centogene mutation database (CentoMD), and ClinVar (https://www.ncbi.nlm.nih.gov/clinvar/ (accessed on 20 October 2020)). To identify the potential disease-causing variant, all relevant information concerning inheritance patterns, family history, and medical records was used to evaluate and identify the variants. Genetic variations related to the index patient’s medical diagnosis were the only ones reported.

### 2.5. Sanger Sequencing

The detected SH3PXD2B variant (c.280C>G) was Sanger sequenced for all five family members being investigated using standard methods (Centogene GmbH, Rostock, Germany).

## 3. Results

### Identification of Missense Mutation in SH3BPXD2B

In this study, we performed W.E.S. to determine the genetic cause underlying the index patient’s clinical presentation. Bioinformatic analysis revealed that the likely pathogenic SH3PXD2B variant is potentially associated with the disease phenotype according to the recommendations of ACMG. One son and both the father and mother in the patient’s family were heterozygous (i.e., carriers). In contrast, the index case was the only known homozygous family member for this variant (Table 2). The variant is a missense point mutation, c.280C>G, causing an amino acid to change from arginine to glycine at position 94 p (Arg94Gly). This is a likely pathogenic variant that has been observed once before and is assumed to be the cause of FTHS [7].

## 4. Discussion

**Figure 3:**
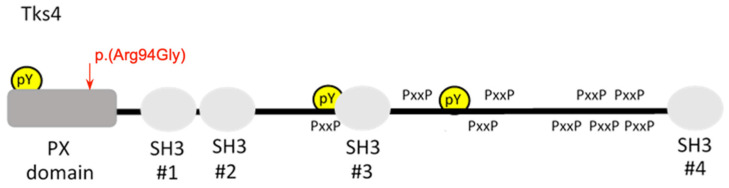
The structure of Tks4 is shown. Dark gray boxes denote P.X. domains, and light gray boxes are SH3 domains. Proline-rich motifs are shown as PxxP and phosphotyrosines as pY. The orange arrow signifies the location of the detected variant.

FTHS is a recessive hereditary disease caused by mutations of the SH3PXD2B (SH3 and PX domains 2B) gene on chromosome 5q35; this gene codes for the TKS4 podosome adaptor protein, which regulates the epidermal growth factor signaling pathway [2,11]. Table 2 summarizes all SH3PXD2B variants reported to be associated with the FTHS phenotype. The primary phenotypic characteristics of FTHS are skeletal, cardiovascular, ocular, and craniofacial anomalies, including brachycephaly, hypertelorism, anterior fontanelle, and developmental delay [1,2]. In this study, many of the index patient’s clinical features and symptoms aligned with FTHS pathogenesis. In addition, W.E.S. analysis of the index patient revealed a homozygous variant in the SH3PXD2B gene that appears to be FTHS-related. This variant is a missense mutation, c.280C>G, causing a substitution of arginine for glycine at position 94 p. (Arg94Gly), as seen in Figure 3. This likely pathogenic variant was previously observed once in another Arab male with FSTH. It is noteworthy to mention that he belongs to consanguineous parents and has a family history of a similar condition and phenotype in three deceased cousins [7]. The concept of consanguinity is a prominent phenomenon in Arab countries and is known to increase the incidence of monogenic congenital anomalies. The majority of autosomal recessive diseases shared by Arab populations are likely to exhibit founder mutations. In light of this, observing the same variant in both families suggests that both result from a founder mutation.

The identified variant is located within the PX domain, which is essential for the Tsk4 function. It is involved in the appropriate cellular localization of Tks4 through its binding to specific membrane lipids, as seen in Figure 3. The protein TKS4 plays an essential role in developing actin-rich membrane projections referred to as podosomes and invadopodia. These projections facilitate pericellular proteolysis and cell migration. Bogel et al. (2012) reported the pivotal role of TKS4 in the epidermal growth factor (EGF) signaling cascade. The authors found that the Tks4 protein in EGF-treated cells was tyrosine-phosphorylated and was linked to EGF receptor activation. When the cells were treated with LY294002, a phosphoinositide (P.I.) 3-kinase blocker (i.e., mutations of the phox homology (PX) domain), tyrosine phosphorylation, and protein translocation through the membrane were significantly reduced [11]. They also reported a Tks4 mutant (R43W) in an FTHS patient exhibiting abnormal intracellular expression and lowered phosphoinositide receptor affinity. Therefore, pathogenic mutations in the PX domain are predicted to abolish binding to phospholipids, thus hindering the formation of the Tsk4-TGFR complex and the subsequent tyrosine-phosphorylation cascade. Upon further analysis, the silencing of Tks4 markedly inhibited HeLa cell migration in a Boyden chamber assay in response to EGF, revealing the function of Tks4 in the modulation of EGF-dependent cell migration [11,12,13,14,15,16].

In support, another study demonstrated that Tks4-deficient mice displayed evident skeletal, ocular, and cardiovascular abnormalities, all of which correspond with the symptoms of FTHS [6]. This further establishes the critical role of Tsk4 in developing multiple varieties of tissues. Moreover, the protein’s function in podosome formation has led to the speculation that defects in podosome synthesis may be involved in human developmental impairments [6]. Together, these findings demonstrate an apparent involvement of Tks4 in FTHS pathogenesis and embryonic development. Thus, patients with FTHS and related disorders have defects in the genes involved in Tks4 interaction and/or the podosome-forming genes.

## 5. Conclusions

This study validated a genetic variant associated with FTHS through parental testing and segregation analysis. The homozygosity of the variant was confirmed; both unaffected parents were heterozygous carriers of SH3PXD2B variant c.280C>G p (Arg94Gly). The variant was also detected in the heterozygous form in the patient’s sibling. This case, which exhibits a unique variant, may aid in the future understanding of FTHS and be added to the ever-growing spectrum of mutations associated with this disorder.

## Figures and Tables

**Table 1:** Clinical features of index patient with the c.280C>G p. (Arg94Gly) SH3PXD2B variant.

Features	Patient V-5
Age	0.33 years
Gender	Male
Weight	4.78 kg
Height	55 cm
Consanguinity	Yes
Cardiovascular pathology	CHD in the form of severe CoA versus I.A.A., small aortic arch, P.D.A., V.S.D., small parachute-like mitral valve
Clinical diagnosis	Frank–Ter Haar syndrome
Neurological abnormalities	I.V.H., mild superior cerebellar vermis atrophy, wide fontanel, bilateral glaucoma
Motor development	Limb abnormalities, dysmorphic features
Other	Low-set ears, congenital glaucoma, dysmorphic features, GERD, talipes, choroid plexus cysts, portal vein thrombosis, gallbladder stone

Abbreviations: I.A.A., interrupted aortic arch; P.D.A., patent ductus arteriosus; V.S.D., ventricle septal defect; CHD, congenital heart disease; I.V.H., intraventricular hemorrhage; GERD, gastroesophageal reflux disease.

**Table 2:** Summary of SH3PXD2B mutations reported in the FTHS literature.

SH3PXD2B Variants	Mutation	Inheritance	Number and Gender of Patients	Heritage of Patient	Clinical Diagnosis	Reference
c.280C>G:p. (Arg94Gly)	Missense point mutation	Autosomal recessive	1 Male	Saudi	Wide fontanel, proptosis, low-set ears, talipes, hypertelorism, synophrys, lower limb abnormalities, right finger anomalies, small head, valve anomalies, severe coarctation, P.D.A., V.S.D., mild superior cerebellar vermis atrophy, congenital bilateral glaucoma	This study
	Novel missense point mutation (first finding)	Autosomal recessive	1 Male	Arab	Megalocornea, congenital cataract, congenital glaucoma, failure to thrive, GDD. The patient’s parents are consanguineous with a family history of a similar condition in 3 deceased cousins with a similar phenotype	[7]
c.578delG, p.S193fs*48	Novel frameshift variant	Autosomal recessive	1 Female	Turkish	Prominent forehead, brachycephaly, wide anterior fontanel, proptosis, hypertelorism, full cheeks, anteverted nostrils, broad mouth, kyphoscoliosis, short hands, camptodactyly, toe anomalies, clubfeet, congenital glaucoma, megalocornea	[8]
c.969delG, p(Arg324Glyfs*19)	Variant altering the formation of podosomes and invadopodia	Autosomal recessive	1 Female	Armenian	IUGR, hypotonia, congenital glaucoma, caudal appendix, scoliosis, camptodactyly, VSD, corpus callosum abnormality, craniofacial defects, buphthalmos, respiratory failure	[1]
chromosome 5q35.1	Loss of function variant (exon 13 deletion)	Autosomal recessive	2 Males and 1 Female	Pakistani	Prominent forehead, hypertelorism, brachycephaly, prominent ears, flat nasal bridge, full cheeks, hypoplasia of the teeth, broad mouth	[5]
chromosome 5q35.1	Insertion at c.147insT, nonsense variant Deletion c.969delG, nonsense variant (p.G323fsX19) Missense variant c.129C>T (p.R43W) Splice-altering variant c.76-2A>C	Autosomal recessive	1 Male	Dutch	Motor retardation, brachycephaly, prominent forehead, hypertelorism, wide anterior fontanels, prominent eyes, congenital glaucoma, large cornea, full cheeks, broad mouth, prominent coccyx, shorthands, megalocornea, clubfeet, anteverted nostrils, thoracolumbar kyphosis, heart murmur, flexion deformity of fingers	[6]
			3 Males	N.R.		[3]
			1 Male and 1 Female	N.R.		[3]
			3 Males	Arab		[6]
			1 Female	Turkish		[9]
			1 Male	Turkish		[6]
			1 Male	Israeli		[6]
c.1188+1773_2733+6592del	Complete loss of SH3PXD2B 12,583 bp deletion	Autosomal recessive	2 Males	Italian	Thick skin, AC, coarse facial features, osteolysis, gingival hyperplasia, brachydactyly, camptodactyly, MVP, heart failure	[10]
c.401+1G-A	Complete loss of SH3PXD2B Splice-altering variant (Glu134GlufsTer1)	Autosomal recessive	1 Male	Australian	Coarse facial features, brachydactyly, pachyermodactyly, MCP, MVP leading to acute congestive cardiac failure, respiratory failure	[10]
c.969delG; p. (Arg324Glyfs*19)	1 bp deletion, frameshift variant	Autosomal recessive	1 Female	Armenian	G.D.D., dysmorphic facial features, brachycephaly, prominent subocular folds, bilateral buphthalmos with megalocornea, bilateral congenital glaucoma, hip dysplasia	[1]
c.250C>T(p.R84*)	Nonsense substitution is predicted to cause premature truncation of the protein coded by the SH3PXD2B gene	Autosomal recessive	2 Males	South Indian	Dysmorphic facial features, brachycephaly, pectus carinatum, kyphoscoliosis, megalocornea, MCP, congenital talipes equinovarus, prominent spine scapula	[2]

Abbreviations: GDD, global developmental delay; VSD, ventricle septal defect; IUG, intrauterine growth retardation; AC, acne conglobate; MVP, mitral valve prolapse; MCP, flexion deformity of the metacarpal joints; N.R., not reported.

## Data Availability

Not applicable.

## Abbreviations

FTHS	Frank–Ter Haar syndrome
IAA	Interrupted aortic arch
PDA	Patent ductus arteriosus
VSD	Ventricle septal defect
CHD	Congenital heart disease
IVH	Intraventricular hemorrhage
GERD	Gastroesophageal reflux disease
CoA	Coarctation of the aorta
MVP	Mitral valve prolapse
